# A simple and versatile fluorochrome‐based procedure for imaging of lipids in arbuscule‐containing cells

**DOI:** 10.1111/tpj.15934

**Published:** 2022-08-29

**Authors:** Héctor Montero, Uta Paszkowski

**Affiliations:** ^1^ Crop Science Centre, Department of Plant Sciences University of Cambridge Cambridge CB3 0LE UK; ^2^ Present address: Molecular Plant Physiology and Biophysics, Julius-von-Sachs-Institute University of Wuerzburg Wuerzburg D-97082 Germany

**Keywords:** arbuscular mycorrhizal symbiosis, lipids, confocal microscopy, arbuscule‐containing cells, cell biology, technical advance

## Abstract

The arbuscular mycorrhizal (AM) symbiosis is characterized by the reciprocal exchange of nutrients. AM fungi are oleaginous microorganisms that obtain essential fatty acids from host plants. A lipid biosynthesis and delivery pathway has been proposed to operate in inner root cortex cells hosting arbuscules, a cell type challenging to access microscopically. Despite the central role lipids play in the association, lipid distribution patterns during arbuscule development are currently unknown. We developed a simple co‐staining method employing fluorophore‐conjugated Wheat Germ Agglutinin (WGA) and a lipophilic blue fluorochrome, Ac‐201, for the simultaneous imaging of arbuscules and lipids distributed within arbuscule‐containing cells in high resolution. We observed lipid distribution patterns in wild‐type root infection zones in a variety of plant species. In addition, we applied this methodology to mutants of the *Lotus japonicus* GRAS transcription factor RAM1 and the *Oryza sativa* half‐size ABC transporter STR1, both proposed to be impaired in the symbiotic lipid biosynthesis‐delivery pathway. We found that lipids accumulated in cortical cells hosting stunted arbuscules in *Ljram1* and *Osstr1*, and observed lipids in the arbuscule body of *Osstr1*, suggesting that in the corresponding plant species, RAM1 and STR1 may not be essential for symbiotic lipid biosynthesis and transfer from arbuscule‐containing cells, respectively. The versatility of this methodology has the potential to help elucidate key questions on the complex lipid dynamics fostering AM symbioses.

## Significance Statement

Lipids play essential roles in the arbuscular mycorrhizal symbiosis, but the study of lipid dynamics in the association has been largely devoid of imaging techniques. A simple and versatile methodology for imaging of lipids in cells containing arbuscules using confocal microscopy was developed. Its implementation allowed unprecedented visualization of diverse patterns of lipid distribution across arbuscule development stages in different plant species and in mutants of genes described to participate in symbiotic lipid dynamics.

## INTRODUCTION

Land plants have engaged with arbuscular mycorrhizal (AM) fungi for millions of years to give rise to one of the most widespread symbioses. The AM association is characterized by the formation of tree‐shaped arbuscules inside root cortical cells, which are the primary symbiotic structures for nutrient exchange. These develop rapidly and their emergence involves important alterations in cellular homeostasis. While AM fungi provide mineral nutrients and water to host plants, they receive carbon in the form of sugars and lipids in return. During arbuscule formation, the periarbuscular membrane (PAM) of plant origin surrounds arbuscule branches, and an apoplastic periarbuscular space (PAS) arises between the PAM and the fungal cell wall. This symbiotic interface is rich in distinctive plant and fungal membrane compartments (Ivanov et al., 2019; Roth et al., 2019) that likely provide the intimate physical contact and magnified surface needed for the efficient exchange of nutrients. The branch domain of the PAM is a specialized membrane that contains several nutrient transporters, supporting the notion of arbuscule‐containing cells being at the core of symbiotic nutrient exchange (Breuillin‐Sessoms et al., 2015; Harrison et al., 2002; Kobae et al., 2010; Kobae & Hata, 2010; Koegel et al., 2013; Pumplin & Harrison, 2009; Tamura et al., 2012).

Arbuscules are ephemeral structures. At the start of arbuscule degeneration, cellular contents are emptied from arbuscule branches followed by hyphal septation, collapse of AM fungal cell walls, and progressive reduction in arbuscule size and organization (Bonfante‐Fasolo, 1984). Arbuscule collapse concurs with the appearance of lipid droplets in host cells (Kobae et al., 2014). Hallmarking mature stages of the symbiosis, lipid‐packed globular fungal structures termed vesicles are formed inside roots (Jabaji‐Hare et al., 1984) and daughter spores develop in the rhizosphere. These spores are carriers of the AM fungal genetic material, but also contain reserves of energy in the form of lipids that will fuel hyphal germination needed to start new cycles of infection.

The AM fungi biomass is primarily oleaginous in nature, and recent developments in AM symbiosis research point towards a pivotal role of lipids in the interaction. Comparative genome‐wide surveys have established that AM fungi lack genes encoding enzymes for *de novo* fatty acid biosynthesis (Morin et al., 2019; Wewer et al., 2014). Therefore, AM fungi are auxotrophs that rely on fatty acids supplied by the host (Jiang et al., 2017; Keymer et al., 2017; Luginbuehl et al., 2017). A recently described pathway for lipid nourishment to the AM fungus has been proposed to operate in arbuscule‐containing cells. This lipid biosynthesis‐delivery pathway is regulated by the GRAS transcription factor REQUIRED FOR ARBUSCULAR MYCORRHIZA 1 (RAM1) and members of the APETALA 2 (AP2) family of transcription factors (Gobbato et al., 2012; Jiang et al., 2018; Luginbuehl et al., 2017; Park et al., 2015; Rich et al., 2021; Xue et al., 2018). Downstream, fatty acid biosynthesis enzymes that operate in arbuscule‐containing cells include the acyl‐ACP thioesterase FatM, the β‐keto‐acyl ACP synthase I (KASI) DISORGANIZED ARBUSCULES (DIS) and the glycerol‐3‐phosphate acyl transferase (GPAT) REQUIRED FOR ARBUSCULAR MYCORRHIZA 2 (RAM2). The fatty acid products of these enzymes have been hypothesized to be transferred to the PAS by the PAM‐localized half‐size ABC transporters STUNTED ARBUSCULE1 (STR1) and STR2 (Brands et al., 2018; Bravo et al., 2017; Jiang et al., 2017; Jiang et al., 2018; Keymer et al., 2017; Luginbuehl et al., 2017; Zhang et al., 2010). Although the components of this pathway have been elucidated in angiosperm model species, recent work in liverworts has demonstrated its broad evolutionary conservation (Rich et al., 2021).

Despite the central role lipids have in the AM association, we do not know the spatial and temporal changes they may experience during the formation of the arbuscules. Visualizing lipids in arbuscule‐containing cells would be useful for the study of these fundamental biological processes in AM symbioses. However, there are limited resources available. The lipophilic dyes Nile red and BODIPY 493/503 have been used to stain extraradical AM structures (Bago et al., 2000; Saito et al., 2004; Sugiura et al., 2020). These dyes have also been used to stain AM fungal structures inside roots or thalli with limited resolution, or also requiring the need of tissue sectioning (Kobae et al., 2014; Luginbuehl et al., 2017; Rich et al., 2021). Nile red has a broad emission range in the orange and red regions of the visible spectrum, while BODIPY dyes fluoresce in the green to orange region with overlapping absorption in relation to other fluorophores. These features may cause difficulties when distinguishing signal from autofluorescence and also hinder co‐localization experiments. In order to study lipid distributions in arbuscule‐containing cells, we developed and applied a method for the simultaneous imaging of lipids and AM fungal chitinaceous cell wall in arbuscule‐containing cells employing the blue lipophilic dye Ac‐201 and Wheat Germ Agglutinin (WGA). Additionally, we applied this new protocol to investigate lipid occurrence and cellular distribution in plant mutants compromised in fatty acid nourishment of the AM fungus observing unexpected patterns of lipid distributions in the mutants.

## RESULTS

To visualize lipids in arbuscule‐containing cells, we tested a blue lipophilic dye, Ac‐201. This dye belongs to the group of amino‐substituted‐trifluoro‐phthalimides, and it was first developed as a lipid droplet‐binding compound in human cancer cells (Puskas et al., 2010). Later, Ac‐201 was used as a marker for lipid droplets in suspension culture cells of diverse plant species and in *Arabidopsis thaliana* germinating seedlings, including roots and shoots (Kuntam et al., 2015). Ac‐201 is a photostable fluorochrome excitable with violet laser and with emission in the blue range (Kuntam et al., 2015). Ac‐201 in conjunction with fluorophore‐conjugated WGA was employed for the simultaneous visualization of AM fungal arbuscules and lipids in arbuscule‐containing cells by using confocal laser‐scanning microscopy (CLSM). While Ac‐201 is here used for the purpose to stain lipids associated with fungal structures for the first time, WGA is a lectin binding to the monomeric unit of chitin, *N*‐acetylglucosamine, and has been extensively used for staining of AM fungal cell walls (Vierheilig et al., 2005). The protocol here established is simple and largely follows the standard WGA‐staining protocol with the omission of initial ethanol incubation and adding an Ac‐201 incubation at the end of the procedure. The detailed steps can be found in the *Experimental Procedures* section. As Ac‐201 is a blue fluorophore, we chose WGA conjugated with the far‐red fluorophore Alexa Fluor™ 633. These blue and far‐red fluorochromes are positioned at opposite extremes of the visible spectra, and have non‐overlapping excitation and emission properties. Throughout this article we provide composite images where the blue channel has been false‐colored into gray for better clarity.

**Figure 1:**
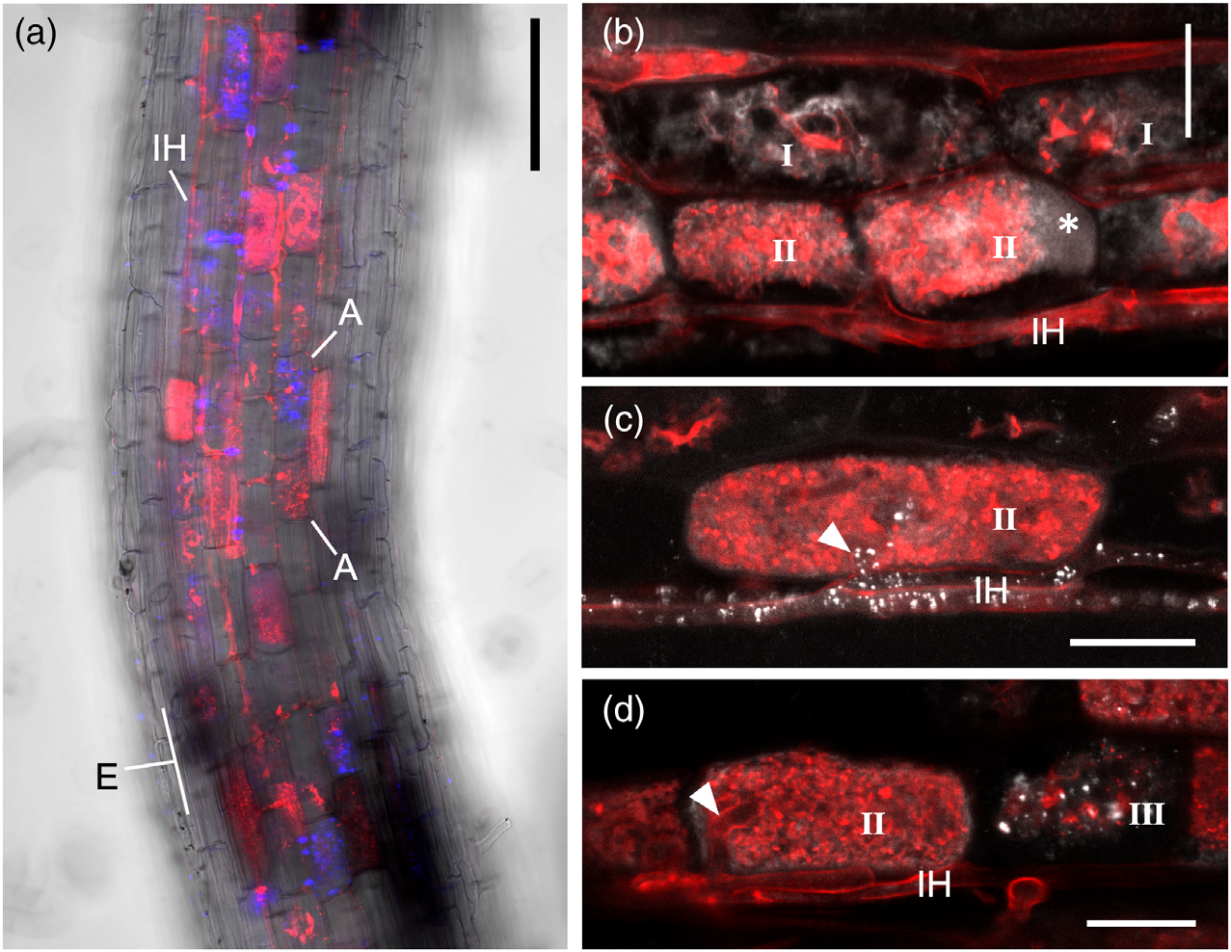
Diversity of lipid distributions in arbuscule‐containing cells of arbuscular mycorrhizal (AM) colonized rice. Rice roots were co‐stained with Wheat Germ Agglutinin (WGA)‐Alexa Fluor™ 633 and Ac‐201 at 10 weeks post‐inoculation (wpi). Confocal laser‐scanning microscopy (CLSM) images show whole‐mount preparations of rice large lateral roots. Red channel corresponds to WGA‐Alexa Fluor™ 633 staining of AM fungal cell walls. Blue channel corresponds to lipophilic dye Ac‐201, and has been false‐colored gray for clarity in (b–d) and in subsequent figures. Composite images are presented here, while individual channels are displayed in Figures S3 and S4. (a) Brightfield, WGA‐Alexa Fluor™ 633 and Ac‐201 composite overview image of a well‐colonized large lateral root. Lipids are closely associated to AM fungal structures, and are not visible in non‐colonized zones such as the root epidermis (E). Inner cortex cells host arbuscules (A) transiting different developmental stages, which is mirrored by diverse lipid distributions. Two adjacent arbuscule‐containing cells harboring contrasting Ac‐201 signal intensities are indicated. (b) Root cortex area exhibiting arbuscule‐containing cells with heterogeneous lipid distribution patterns. Lower‐order branching arbuscules with lipids distributed closely associated to AM hyphae (I) co‐occur with higher‐order branching arbuscules presenting uniform lipid signal (II). An asterisk marks lipids present in the lumen of an arbuscule‐containing cell. (c) A fully developed arbuscule with uniform distribution of lipids (II) and with presence of lipids in the arbuscule trunk (arrowhead). (d) A fully developed arbuscule with uniform lipid signal (II), albeit absent from the arbuscule trunk (arrowhead), co‐occurring with an adjacent collapsing arbuscule displaying bright Ac‐201 dye signal in small clusters (III). IH, intraradical hyphae. Scale bars: 100 μm (a); 20 μm (b–d). [Colour figure can be viewed at wileyonlinelibrary.com]

We first tested the method in rice (*Oryza sativa*) colonized with the AM fungus *Rhizophagus irregularis*. Blue signal from the lipophilic dye Ac‐201 was evidently associated with AM fungal structures and absent from non‐colonized areas such as the root epidermis (Figure 1a). Ac‐201 was largely undetected in non‐colonized cells from colonized roots or in uninoculated roots, in which Ac‐201 signal could only be seen faintly staining the hydrophobic plant cell walls (Figure S1). As expected, storage AM fungal vesicles, known carriers of lipids, displayed bright Ac‐201 signal (Figure S2). We encountered arbuscule‐containing cells exhibiting great diversity of lipid distribution patterns. In arbuscule‐containing cells hosting lower‐order branching arbuscules, morphologically consistent with young developing arbuscules, it was possible to observe lipids inside hyphae, while in higher‐order branching arbuscules, the pattern changed with lipids being more abundant and uniformly distributed perhaps localizing inside arbuscules and/or around their immediate vicinity. It was additionally possible to discern lipids in the lumen of arbuscule‐containing cells (Figure 1b). While lipids were seen uniformly distributed in cells hosting fully developed arbuscules, it was interesting to see that lipids were frequently present in the arbuscule trunks and surrounding intraradical hyphae (Figure 1b–d). When present, these lipids are arranged in small clusters (Figure 1c). In cells hosting collapsing arbuscules, lipids accumulated in small clusters closely associated with the degenerating arbuscule body (Figure 1d). These observations provide evidence for the dynamically changing lipid distribution accompanying arbuscule development.

**Figure 2:**
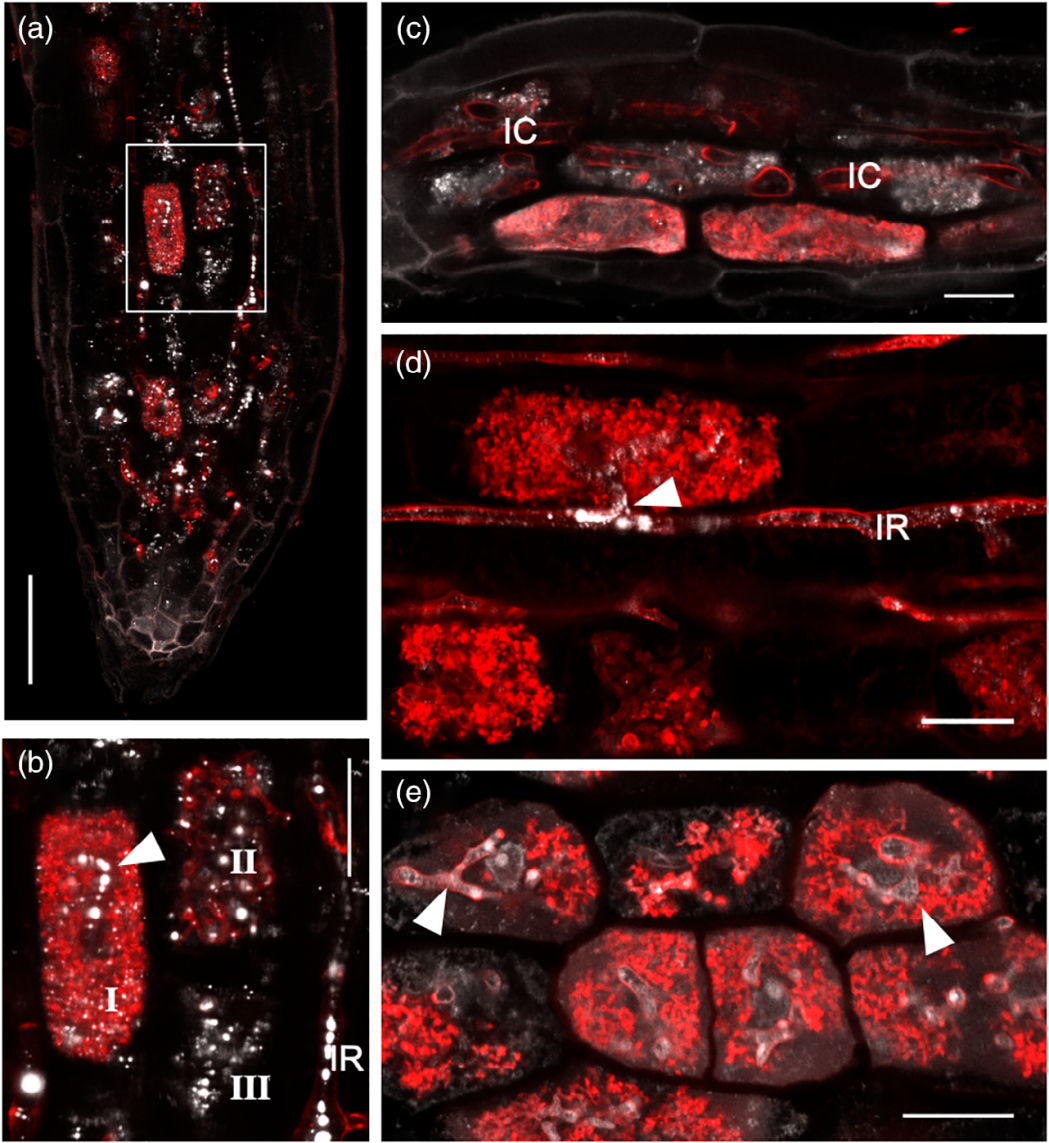
Lipid imaging in different plant species colonized with *Rhizophagus irregularis*. Roots were treated with Wheat Germ Agglutinin (WGA)‐Alexa Fluor™ 633 (red) and Ac‐201 (gray). Confocal laser‐scanning microscopy (CLSM) images are from whole‐mount preparations. Composite images are presented here, while individual channels are displayed in Figure S5. (a) *Zea mays* root colonized at 6 weeks post‐inoculation (wpi). (b) Inset from (a). Lipid accumulation is shown in cells hosting a fully developed arbuscule (I), a collapsing arbuscule (II), and a cell hosting an arbuscule in an advanced stage of collapse (III). (c) *Brachypodium distachyon* root colonized at 6 wpi. Lipids tend to accumulate at arbuscule branches. (d) Root cortex cell of *Lotus japonicus* at 6 wpi hosting a fully developed arbuscule with lipids accumulating in the arbuscule trunk. (e) *Carica papaya* root area colonized at 6 wpi. Filled arrowheads, arbuscule trunks; IC, intracellular hyphae; IR, intraradical hyphae. Scale bars: 50 μm (a); 20 μm (b–e). [Colour figure can be viewed at wileyonlinelibrary.com]

Next, we examined the suitability of the protocol in other plant species inoculated with *R. irregularis*. Similar to the case of rice, maize (*Zea mays*) roots showed lipids associated with cortical cells hosting arbuscules of different developmental stages (Figure 2a,b). In the grass model *Brachypodium distachyon*, *R. irregularis* has been reported to favor the symplastic route for cell‐to‐cell hyphal spread (Hong et al., 2012). As such, this species mostly hosts coarse intracellular hyphae traversing root cortical cells as opposed to arbuscule trunks normally observed in rice or maize. Lipids were observed to accumulate mainly at the arbuscule branches (Figure 2c). We also examined the applicability of the protocol to eudicot plant species. In the legume *Lotus japonicus*, lipids were mainly present in the arbuscule body (Figure 2d). *Carica papaya* belongs to a group of basal AM mycorrhizal species from the Brassicales order, a lineage that has lost the AM mycorrhizal trait in derived taxa such as in the Brassicaceae family (Delaux et al., 2014). Lipids were seen uniformly distributed in arbuscule‐containing cells and abundant in arbuscule trunks (Figure 2e). These observations illustrate that the protocol is useful for a variety of plant species deploying different ways of interacting with AM fungi.

**Figure 3:**
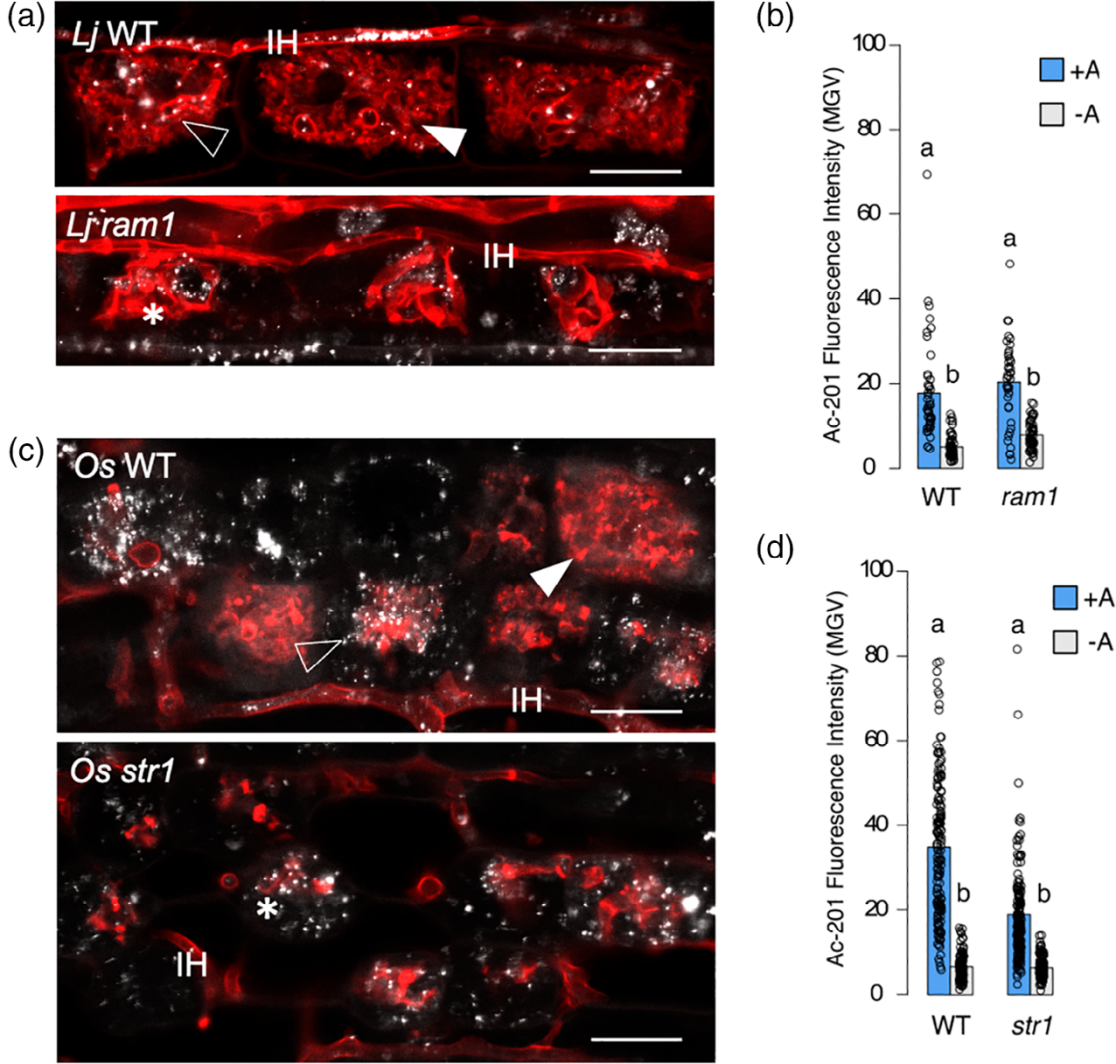
Lipid distributions of mutants of *LjRAM1* and *OsSTR1*. Confocal laser‐scanning microscopy (CLSM) images show whole‐mount root preparations. Composite images are presented here while individual channels are displayed in Figure S6. (a) *Lotus japonicus* roots co‐stained with Wheat Germ Agglutinin (WGA)‐Alexa Fluor™ 633 (red) and Ac‐201 (gray) at 6 weeks post‐planting in nurse plant system. (b) Ac‐201 fluorescence intensity plots of arbuscule‐containing cells (+A) and non‐colonized neighboring cells (−A) from *L. japonicus* Gifu wild‐type and *ram1* mutant roots. Bars represent means. The individual data points display the combined data from three individual plants. In each plant, a range of 13–16 +A cells and 14–17 −A cells were analyzed. Fluorescence intensity is measured as mean gray value (MGV) from grayscale‐converted blue channel CLSM images. Different lowercase letters indicate statistically significant differences (Wilcoxon test, *P* < 0.05). (c) Rice roots co‐stained with WGA‐Alexa Fluor™ 633 (red) and Ac‐201 (gray) at 5 weeks post‐planting in nurse plant system. (d) Ac‐201 fluorescence intensity plots of arbuscule‐containing cells (+A) and non‐colonized neighboring cells (−A) from *Oryza sativa* Nipponbare wild‐type and *str1* mutant roots. Bars represent means. The individual data points display the combined data from three individual plants. In each plant, a range of 41–50 +A cells and 40–44 −A cells were analyzed. Fluorescence intensity is measured as MGV from grayscale‐converted blue channel CLSM images. Different lowercase letters indicate statistically significant differences (Wilcoxon test, *P* < 0.05). Filled arrowhead, mature arbuscule. Unfilled arrowhead, arbuscule‐containing cell with proliferation of lipid droplets. *stunted arbuscule. Scale bar: 20 μm. [Colour figure can be viewed at wileyonlinelibrary.com]

We aimed to test our staining and imaging method using mutants of two genes described to operate in the proposed AM lipid biosynthesis and delivery pathway. The transcription factor RAM1 has been regarded as an important regulator of lipid biosynthesis in arbuscule‐containing cells (Luginbuehl et al., 2017). In *L. japonicus*, the *ram1* mutant phenotype corresponds to severe depression in colonization and stunted arbuscules (Pimprikar et al., 2016; Xue et al., 2015). Likewise, the rice PAM‐localized half‐size ABC transporter STR1 has also a reduced colonization mutant phenotype accompanied by stunted arbuscules (Gutjahr et al., 2012) and has been proposed to function downstream of the pathway delivering lipids to the AM fungus. We selected the *L. japonicus ram1‐3* allele (Pimprikar et al., 2016) and the rice *str1‐2* allele (Gutjahr et al., 2012). We hypothesized an absence of lipids accumulating in arbuscule‐containing cells of the *Ljram1* mutant and an absence of lipids accumulating inside AM fungal arbuscules in the *Osstr1* mutant. In these mutant alleles, arbuscules are seldom present but it has been reported that by employing nurse plant systems, the quantity of colonization increases while stunted arbuscule phenotypes are maintained, prompting us to choose nurse plant systems in our experimental design. As expected, arbuscules were stunted in the two mutants. Contrary to our expectations, in *Ljram1*, lipids were present in arbuscule‐containing cells. They appeared to be closely associated to the stunted arbuscule mass and we did not encounter the situation where lipids were observed inside the arbuscule body (Figure 3a,b). Although stunted arbuscules inhabit a highly disorganized cellular landscape preventing us to unequivocally discern the specific location of the lipids, their sole presence in arbuscule‐containing cells is unexpected considering that RAM1 has been described as directing lipid biosynthesis in arbuscule‐containing cells (Luginbuehl et al., 2017). In *Osstr1*, we found that lipids accumulate in the underdeveloped arbuscules in a pattern resembling collapsing arbuscules of wild‐type plants (Figure 3c,d). As in the case of *Ljram1*, no specific localization can be established for greatly disorganized arbuscules, although occasionally it was possible to encounter lipids evidently localizing inside the stunted arbuscule body (Figure S7), implying that lipid transport from the host cell to the arbuscule is not fully abolished in *Osstr1*. Besides demonstrating that the protocol is useful for the study of mutants, these results show that in the absence of *LjRAM1* or *OsSTR1*, lipids still occur in arbuscule‐containing cells. These observations suggest that complex dynamics underpinning biosynthesis, distribution and transfer of lipids may occur in arbuscule‐containing cells.

## DISCUSSION

Attending to the important role of lipids in the AM symbiosis, we sought to develop a method to visualize lipids in the cells where arbuscules develop. Previously used lipophilic dyes in AM research include Nile red and BODIPY, however mostly for extraradical AM structures or intraradical structures but with limited resolution. Both fluorophores have broad absorption and emission ranges hindering co‐localization studies, and BODIPY has low photo‐stability (Ohsaki et al., 2010). To address this, lipids in arbuscule‐containing cells were observed employing the lipophilic dye Ac‐201, which has close to ultraviolet excitation/emission properties. No biochemical reports have described the specific range of lipids Ac‐201 binds to. However, this dye was shown to co‐localize with Nile red, and therefore is likely to stain a broad range of neutral lipids (Kuntam et al., 2015). The Ac‐201 signal was bright and clearly associated with AM fungal colonized root regions. Besides, the blue Ac‐201 signal was easily distinguishable from the far‐red WGA‐Alexa Fluor™ 633 AM fungal chitin signal. Indeed, because both dyes are positioned at the extremes of the visible spectra, the protocol can potentially be adapted by introducing a third green/yellow dye providing its compatibility with the treatment. The methodology presented here involves KOH‐fixed whole‐mount root preparations. In these conditions, the lipophilic dye Ac‐201 could readily penetrate inner cortex tissues populated by arbuscules in different plant species. Thus, co‐staining AM colonized roots with Ac‐201 and WGA‐Alexa Fluor™ 633 proved to be a useful method to visualize lipids in arbuscule‐containing cells in unprecedented high resolution.

Simultaneous imaging of AM fungal chitin and lipids revealed that arbuscules at comparable developmental stage in a common root area can be hosted by cells harboring lipids in diverse quantities and distribution patterns. Lipids were found in arbuscule trunks, arbuscule branches and in the lumen of the arbuscule‐containing plant cells. Clustering of lipids was observed in senescent arbuscules. This likely reflects that lipid dynamics in arbuscule‐containing cells respond to the rapid and complex processes associated with arbuscule development, where root cells and AM fungi have a limited time window to handle nutritional resources being exchanged.

We applied our protocol to two mutants impaired in constituents of a pathway proposed to be required for AM fungal lipid nourishment; the upstream transcription factor RAM1 and the downstream PAM‐localized transporter STR1. Previous work has shown that disruption of components of this pathway results in lipids not to be transferred to AM fungi and also in alterations in arbuscule morphology (Brands et al., 2018; Bravo et al., 2017; Jiang et al., 2017; Jiang et al., 2018; Keymer et al., 2017; Luginbuehl et al., 2017; Xue et al., 2018). We found that lipids are encountered in host cells with stunted arbuscules of the *Ljram1* and *Osstr1* mutants. These lipids most likely originate from the arbuscule‐containing cells themselves considering that no lipids were observed in neighboring non‐colonized cells. This may imply that in the mutants, a RAM1‐independent lipid biosynthesis pathway operates in arbuscule‐containing cells. Alternatively, lipids associated with arbuscules in the mutants may originate from nurse plants, which might translocate lipids via the common mycorrhizal network. Such mechanisms of nutrient translocation between arbuscules hosted in different plants are currently unknown to exist. The stunted arbuscule phenotype reported for most mutants of the proposed lipid biosynthesis‐delivery pathway cannot be rescued by wild‐type nurse plants. This is the case for *LjRAM2*, *LjDIS*, *LjSTR1* (Keymer et al., 2017), *OsSTR1* (Gutjahr et al., 2012) and *MtFatM* (Jiang et al., 2018). One exception was a mutant of *MtRAM2*, whose stunted arbuscule phenotype was fully (Luginbuehl et al., 2017) or partially (Jiang et al., 2017) rescued by wild‐type nurse plants. Because of these arbuscule morphology phenotypes, at present it is not clear if the proposed pathway is directly involved in fungal nourishment, or indirectly by regulating arbuscule development. Very little is known about the post‐arbuscule development stage of the AM symbiosis where nutrients exchanged are metabolized, leading to subsequent physiological changes. When arbuscule degeneration starts, hyphal septation occurs at their branches and trunks and lipid droplets emerge (Kobae et al., 2014). The means by which arbuscules acquire fatty acids supplied by plants and the fate of the nutritional resources still in the body of a collapsing arbuscule are unknown. They necessarily include an important pool of membrane phospholipids whose metabolic products are not known to either be recycled by the plant or reach the AM fungus. Conciliating these observations and elucidating the mechanism(s) of lipid transfer to the AM fungus remain open questions, and highlight that there is much to learn about lipid dynamics in AM symbiosis. Imaging tools like those presented here are a promising resource for future studies aiming to disentangle complex nutrient dynamics pivotal for AM symbiosis sustenance.

In summary, we have developed a versatile and simple method to visualize in high resolution lipids in arbuscule‐containing cells of whole‐mount root preparations employing the fluorochromes Ac‐201 and WGA‐Alexa Fluor™ 633. This method was easily implemented in all plant species tested and can potentially be used in a variety of contexts to advance the understanding of lipid dynamics, a rising field in AM symbiosis research.

## Experimental Procedures

### Ac 201‐WGA co‐staining

Our methodology for CLSM imaging of lipids in arbuscule‐containing cells employs Ac‐201 and fluorophore‐conjugated WGA co‐staining. Roots were harvested and incubated in 20% (w/v) KOH for 2 days. KOH was removed and roots were rinsed with diH_2_O after which samples were incubated for 2 h in 0.1 m HCl. Roots were rinsed with 1 × phosphate‐buffered saline (PBS; pH 7.4) solution. A 0.2 μg ml^−1^ WGA‐Alexa Fluor™ 633 (Invitrogen, Carlsbad, CA, USA) solution in 1 × PBS was added, and samples were incubated at 4°C in the dark for at least 5 days. Roots treated with WGA were counter‐stained with 5 μg ml^−1^ of Ac‐201 (Avicor, Szeged, Hungary) and incubated at room temperature for 1 h, occasionally inverting the tube for homogenous mixing, after which roots were briefly rinsed in diH_2_O and mounted in microscope slides for imaging.

### Confocal laser‐scanning microscopy

Imaging of roots took place using a Leica TCS SP8 (Leica Microsystems, Wetzlar, Germany). For the simultaneous imaging of fungal chitin and lipids, WGA‐Alexa Fluor™ 633 was detected using white light laser with an excitation wavelength of 630 nm (5.6% laser power), and emitted wavelengths collected at 650–730 nm. To detect Ac‐201, a UV laser was used with an excitation wavelength of 405 nm (5.6% laser power) and emitted wavelengths collected at 410–510 nm. Images were acquired with a line average of 2 and dimension of 2048 × 2048 pixels. Roots were observed using a 40 × water immersion objective. Image processing was carried out using the Fiji package under the ImageJ software license (Schindelin et al., 2012). For measurements of Ac‐201 fluorescence intensity in arbuscule‐containing cells and neighboring non‐colonized cells, blue channel images were converted to grayscale in Fiji, and the freehand selection tool was used to individualize cells. Mean gray values (MGV) were measured on the selection area, corresponding to the sum of the gray values of all pixels in the selection area divided by the number of pixels.

### Plant and fungal material

Wild‐type and *str1‐2* mutant allele (ID: CL522472), generated in Gutjahr et al. (2012), are in *O. sativa* subsp. japonica cv. Nipponbare background. *Lotus japonicus ram1‐3* allele (ID: SL0181) in Gifu background was generated in Pimprikar et al. (2016). Other wild‐type plant species used in this study are *B. distachyon*, inbred line Bd21, *Z. mays* inbred line B73, and *C. papaya* variety Solo Sunrise. The AM fungal model species *R. irregularis* (DAOM197198) was employed for all inoculation assays, and spores were sourced from *Agrobacterium rhizogenes*‐transformed carrot hairy root cultures (Becard & Fortin, 1988). Three‐hundred AM fungal spores were applied to individual germinating seedlings. *Oryza sativa*, *B. distachyon* and *C. papaya* plants were grown in cones (12 cm depth, 3 cm diameter). *Zea mays* were grown in pots (10 cm diameter). *Oryza sativa* nurse plant system consisted of individual wild‐type and mutant plants growing in common black Petri dishes (4 cm diameter). *Lotus japonicus* nurse plant system consisted of two wild‐type and two mutant plants growing in common pots (10 cm diameter). Sand substrate was employed for *O. sativa* and *Z. mays*. Substrate for *B. distachyon*, *C. papaya* and *L. japonicus* consisted of a 2:1 mixture of sand and Terra‐Green®. *Oryza sativa*, *Z. mays* and *C. papaya* colonization experiments were carried out in a phytochamber with a photoperiod of 12‐h day–night cycle at a fluctuating temperature of 28/20°C day–night and 65% relative humidity under fluorescent lamp illumination with a light intensity of 400 μmol μm^−2^ sec^−1^. Colonization assays of *B. distachyon* and *L. japonicus* were carried out in a phytochamber with photoperiod and temperature conditions of 16/8‐h day–night cycle at constant 20°C and 60% relative humidity under a light intensity of 150 μmol μm^−2^ sec^−1^. Plants were watered every second day, the first 2 weeks post‐inoculation (wpi) with reverse osmosis water followed by a low phosphate fertilization regime consisting of half‐strength Hoagland solution containing 25 μm KH_2_PO_4_ every other watering day.

## AUTHOR CONTRIBUTIONS

HM and UP designed research; HM performed the experiments; HM and UP wrote the manuscript.

## CONFLICT OF INTEREST

The authors have no conflicts of interest.

## Supporting information


**Figure S1.** Lipid distribution in non‐colonized cortex cells and in uninoculated roots.
**Figure S2.** Lipid distribution in AM fungal vesicles.
**Figure S3.** Detail of Figure 1(a) displaying individual CLSM channels and overlays.
**Figure S4.** Detail of Figure 1(b–d) displaying individual CLSM channels and overlays.
**Figure S5.** Detail of Figure 2 displaying individual CLSM channels and overlays.
**Figure S6.** Detail of Figure 3 displaying individual CLSM channels and overlays.
**Figure S7.** Lipid distribution in *Osstr1* mutant.

## Data Availability

All data generated or analyzed during this study are included in this published article and its supplementary information files.
