# Supplementary material for: A simple and versatile fluorochrome‐based procedure for imaging of lipids in arbuscule‐containing cells
Source: Plant J. 2022 Aug 29;112(1):294–301. doi: 10.1111/tpj.15934 (PMC9804681; doi:10.1111/tpj.15934)
Supplement: Supplementary file 1 — Figure S1. Lipid distribution in non‐colonized cortex cells and in uninoculated roots. Figure S2. Lipid distribution in AM fungal vesicles. Figure S3. Detail of Figure 1(a) displaying individual CLSM channels and overlays. Figure S4. Detail of Figure 1(b–d) displaying individual CLSM channels and overlays. Figure S5. Detail of Figure 2 displaying individual CLSM channels and overlays. Figure S6. Detail of Figure 3 displaying individual CLSM channels and overlays. Figure S7. Lipid distribution in Osstr1 mutant. [file TPJ-112-294-s001.docx]

## Supporting Information

Article title: **A simple and versatile fluorochrome-based procedure for imaging of lipids in arbuscule-containing cells**

Authors: **Héctor Montero and Uta Paszkowski**

The following Supporting Information is available for this article:

**Figure S1. Lipid distribution in non-colonized cortex cells and in uninoculated roots**

**Figure S2. Lipid distribution in AM fungal vesicles**

**Figure S3. Detail of Fig.1a displaying individual CLSM channels and overlays**

**Figure S4. Detail of Fig.1b-d displaying individual CLSM channels and overlays**

**Figure S5. Detail of Fig.2 displaying individual CLSM channels and overlays**

**Figure S6. Detail of Fig.3 displaying individual CLSM channels and overlays**

**Figure S7. Lipid distribution in *Osstr1* mutant**


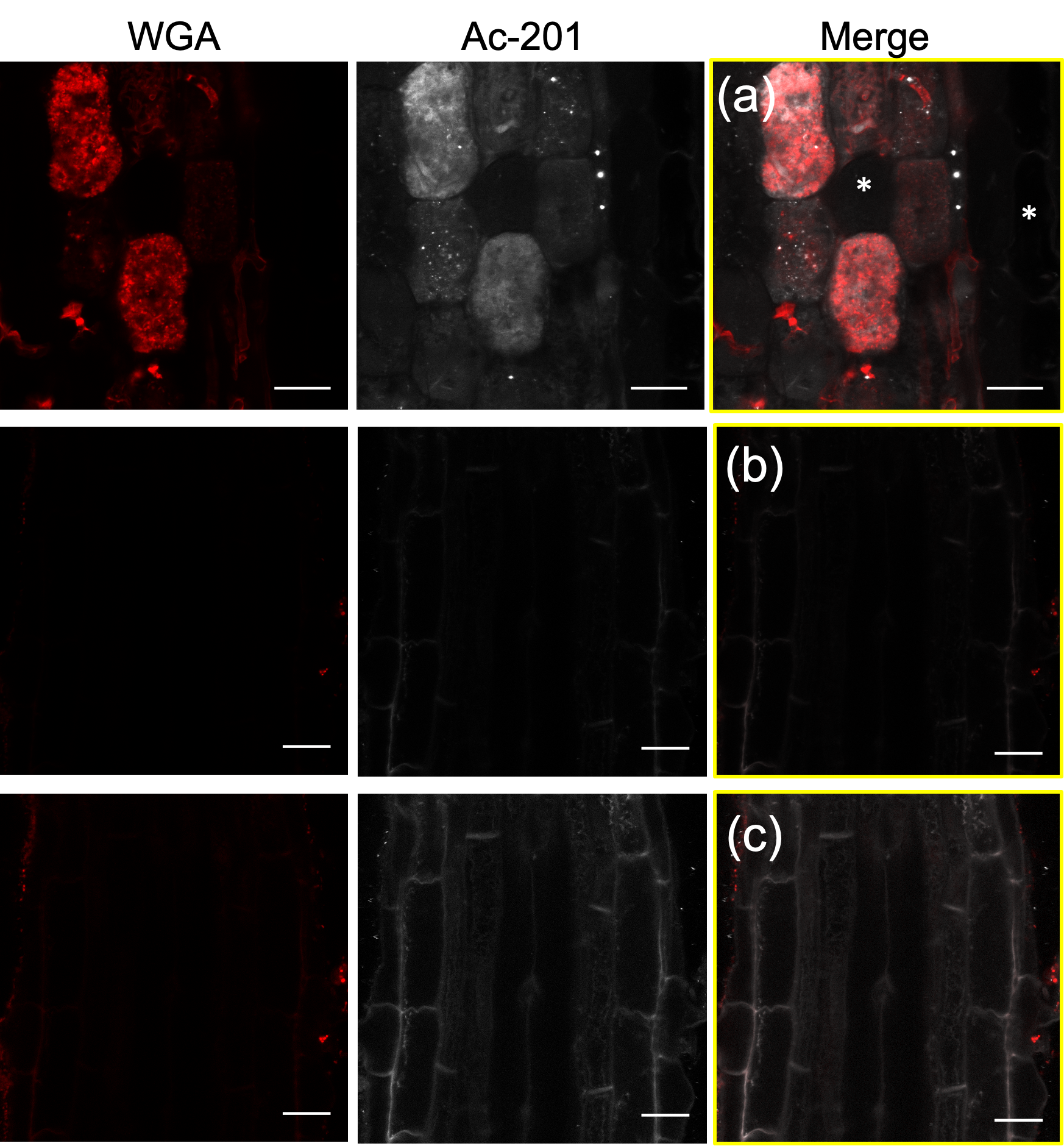


**Figure S1**: Lipid distribution in non-colonized cortex cells and in uninoculated roots. CLSM images from whole-mount preparations of rice large lateral roots co-stained with WGA-Alexa Fluor™ 633 (red) and Ac-201 (grey). (a) AM fungal structures in inoculated rice root. Asterisks mark cortex cells that are not hosting AM fungi and that harbour no Ac-201 signal (b) Root from uninoculated plant displaying faint cell wall autofluorescence. (c) Same image as (b) with 60% increased brightness in all channels. Scale bar, 20 µm.


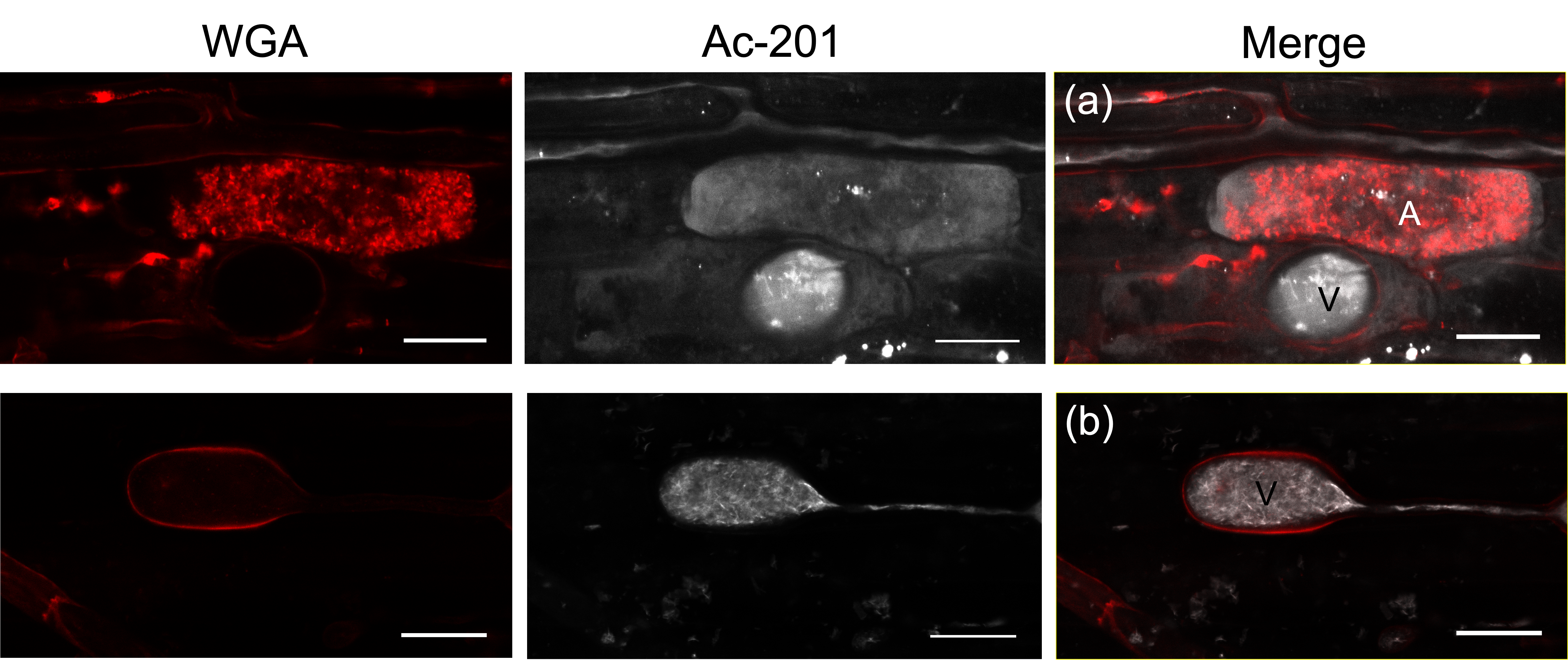


**Figure S2**: Lipid distribution in AM fungal vesicles. CLSM images from AM fungal vesicles co-stained with WGA-Alexa Fluor™ 633 (red) and Ac-201 (grey). (a) Intraradical AM fungal vesicle (V) located adjacent to an arbuscule-containing cell (A). (b) A free AM fungal vesicle. Scale bar, 20 µm.


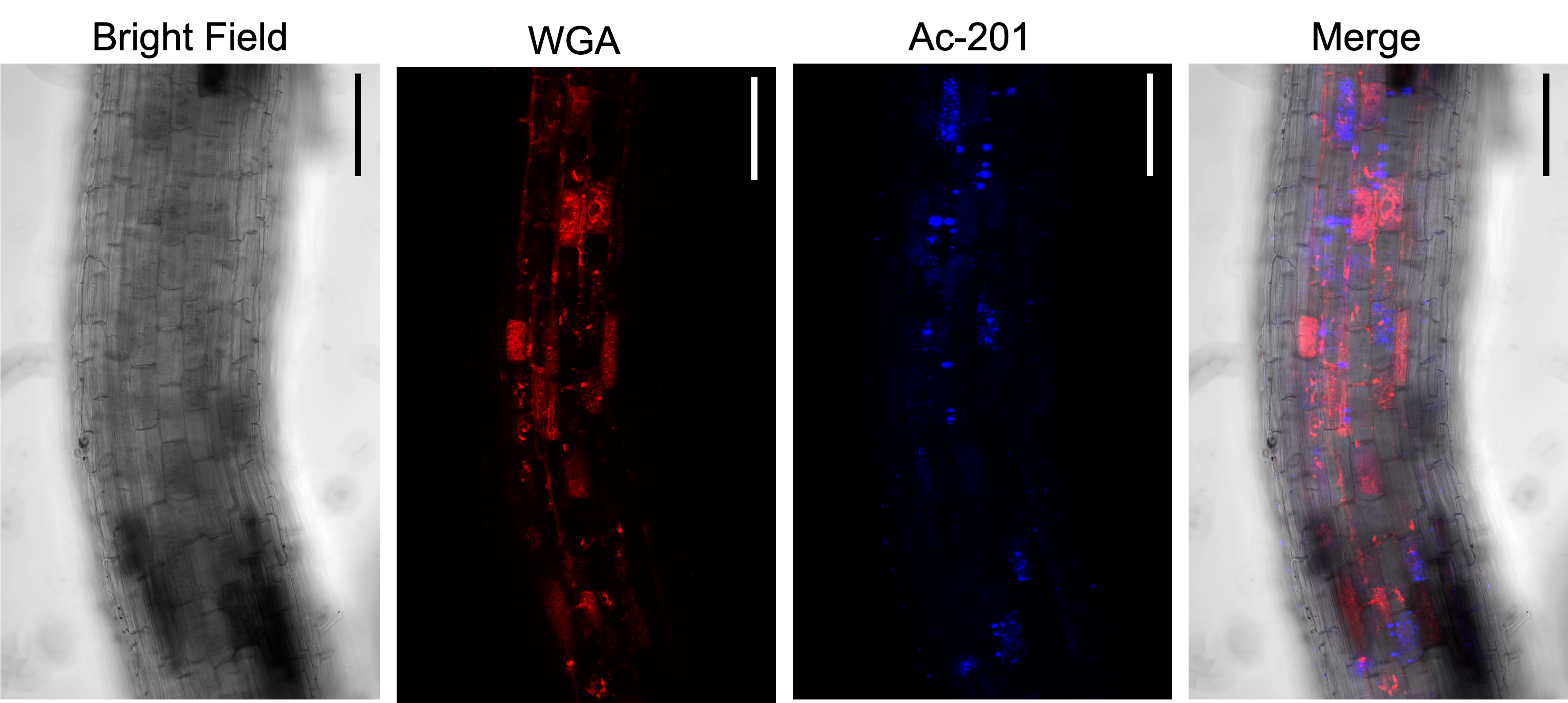


**Figure S3**: Detail of Fig.1a displaying individual CLSM channels and overlays. Diversity of lipid distributions in arbuscule-containing cells of AM colonized rice. Rice roots were co-stained with WGA-Alexa Fluor™ 633 and Ac-201 at ten weeks post-inoculation. CLSM image shows a whole-mount preparation of a well colonized rice large lateral root. Red channel corresponds to WGA-Alexa Fluor™ 633 staining of AM fungal cell walls. Blue channel corresponds to lipophilic dye Ac-201. Scale bar, 100 µm.


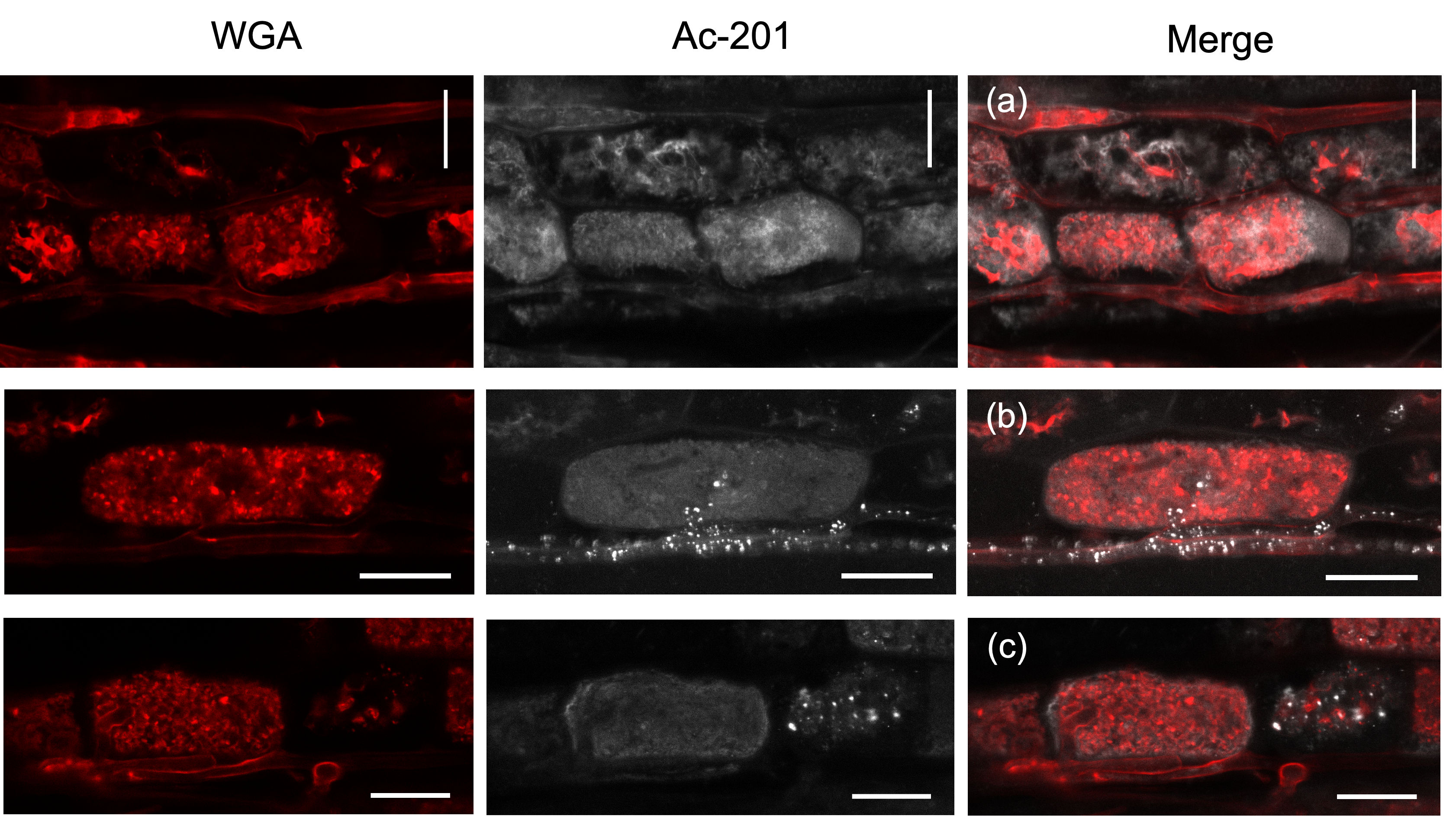


**Figure S4**: Detail of Fig.1b-c displaying individual CLSM channels and overlays. Diversity of lipid distributions in arbuscule-containing cells of AM colonized rice. CLSM image shows whole-mount preparations of rice large lateral roots. Red channel corresponds to WGA-Alexa Fluor™ 633. Blue channel corresponds to Ac-201. Blue channel has been in addition false-colored grey. (a) Root cortex area exhibiting arbuscule-containing cells with heterogeneous lipid distribution patterns. (b) A fully developed arbuscule with uniform distribution of lipids and with presence of lipids in arbuscule trunk. (c) A fully developed arbuscule with uniform lipid signal, albeit absent form arbuscule trunk co-occurring with an adjacent collapsing arbuscule displaying bright Ac-201 dye signal in small clusters. Scale bar, 20 µm.


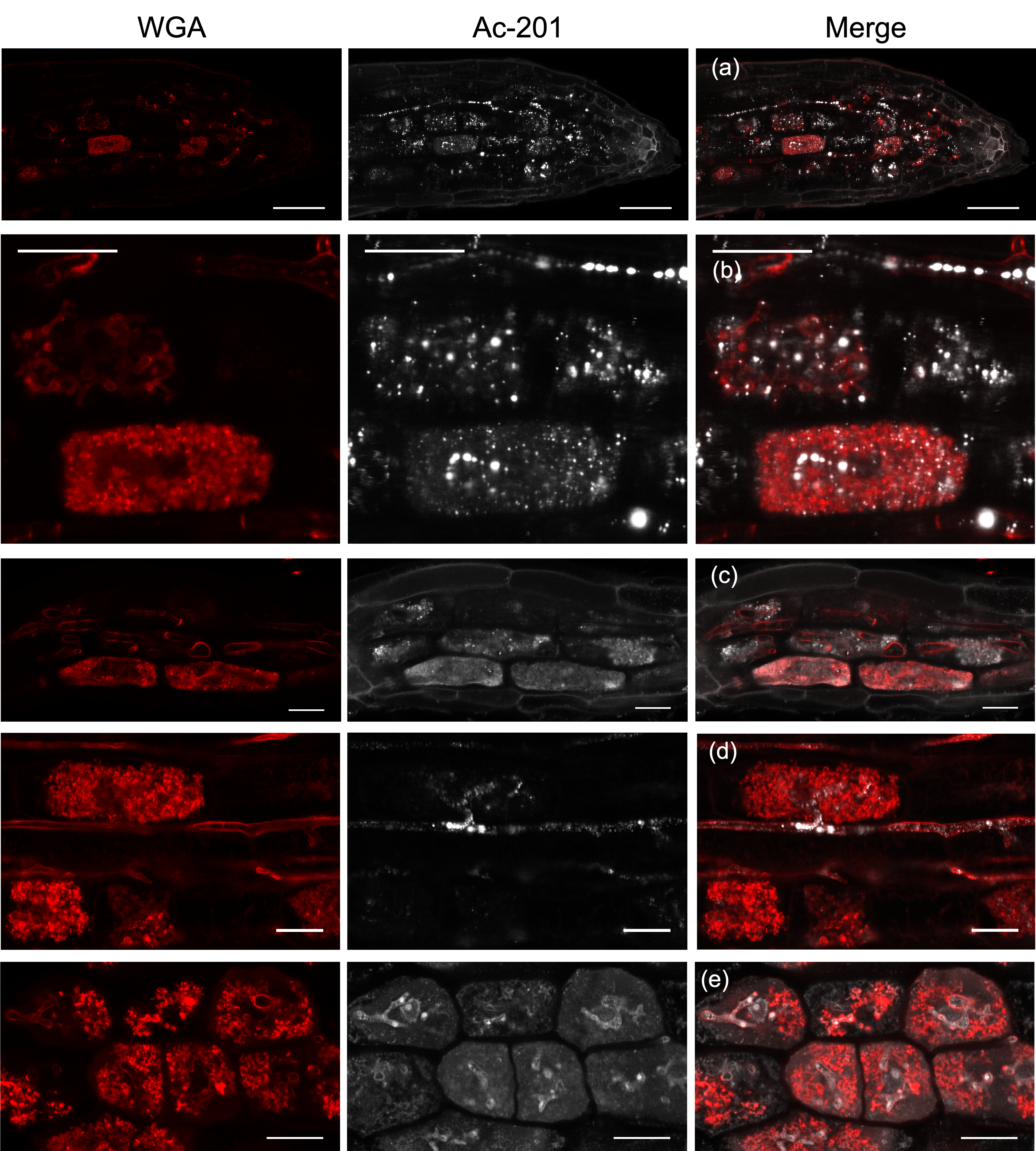


**Figure S5**: Detail of Fig.2 displaying individual CLSM channels and overlays. Lipid imaging in different plant species colonized with *R. irregularis*. Roots were treated with WGA-Alexa Fluor™ 633 (red) and Ac-201 (grey). CLSM images are from whole-mount preparations. (a) *Z. mays* root colonized at six weeks post-inoculation. (b) Inset from (a). (c) *B. distachyon* root colonized at six weeks post-inoculation. (d) Root cortex cell of *L. japonicus* at six weeks post-inoculation. (e) *C. papaya* root area colonized at six weeks post-inoculation. Scale bar subfigure (a), 50 µm. Scale bar subfigures (b-e), 20 µm.


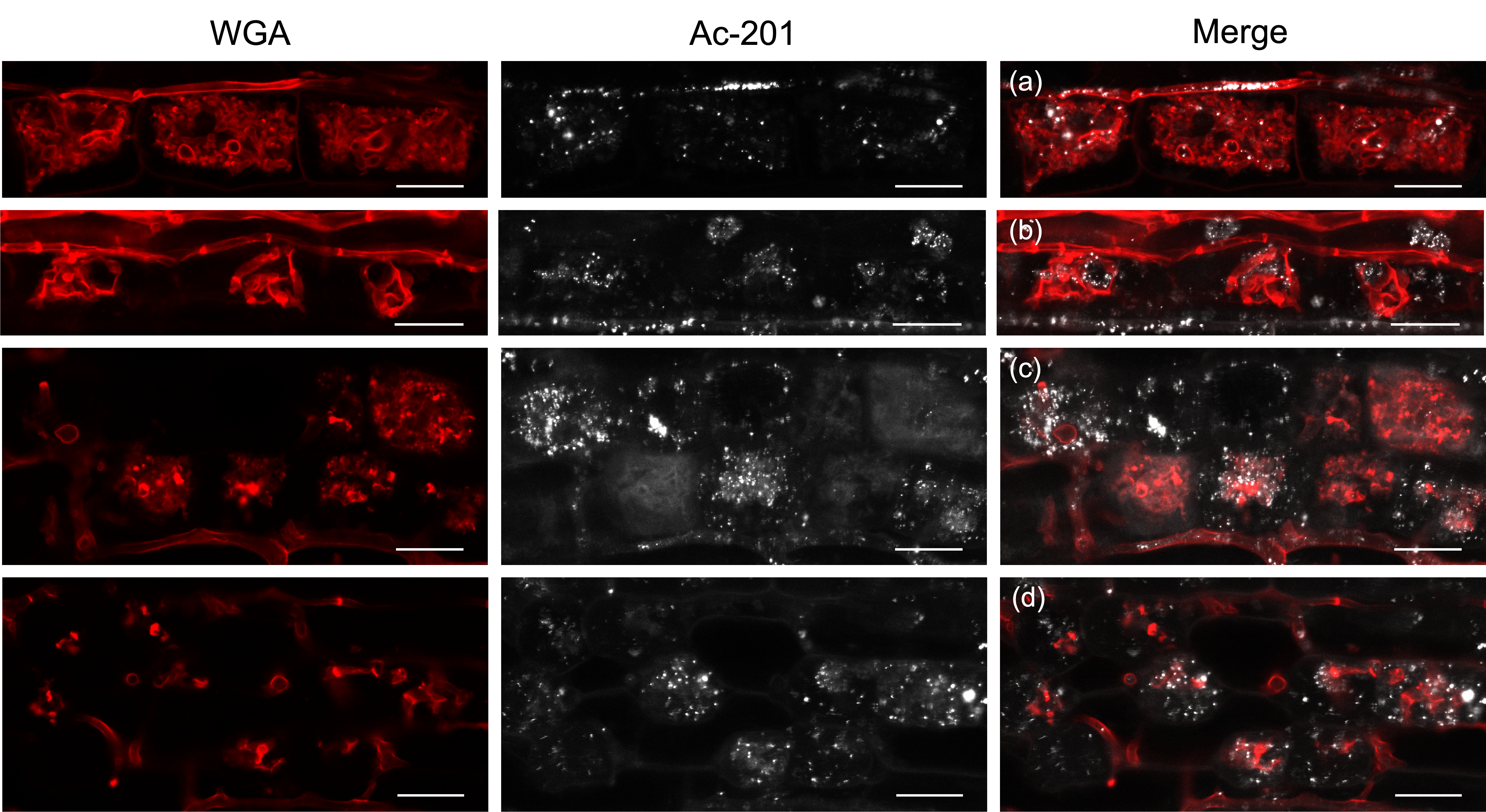


**Figure S6**: Detail of Fig.3 displaying individual CLSM channels and overlays. Lipid distributions of mutants of *LjRAM1* and *OsSTR1*. Roots were treated with WGA-Alexa Fluor™ 633 (red) and Ac-201 (grey). CLSM images show whole-mount root preparations. (a) *L. japonicus* wild-type. (b) *L. japonicus* *ram1* mutant. (c) Rice wild-type. (d) Rice *str1* mutant. Scale bar, 20 µm.


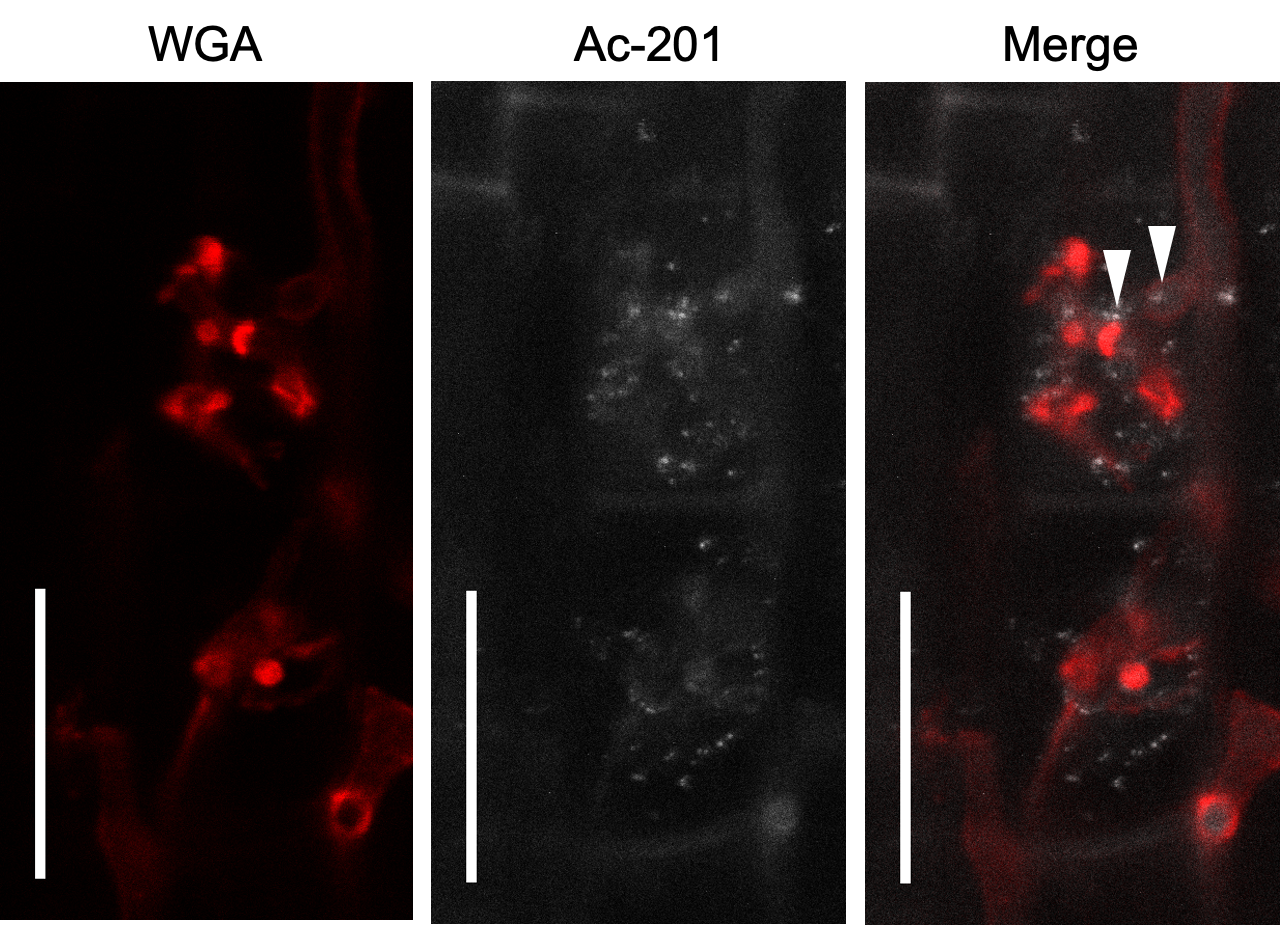


**Figure S7**: Lipid distribution in *Osstr1* mutant. CLSM image from stunted arbuscules of *Osstr1* mutant co-stained with WGA-Alexa Fluor™ 633 (red) and Ac-201 (grey). Arrows point to lipids localizing inside arbuscule body. Scale bar, 20 µm.
